# The impact of drug resistance on the risk of tuberculosis infection and disease in child household contacts: a cross sectional study

**DOI:** 10.1186/s12879-017-2668-2

**Published:** 2017-08-29

**Authors:** Vera Golla, Kathryn Snow, Anna M. Mandalakas, Simon H. Schaaf, Karen Du Preez, Anneke C. Hesseling, James A. Seddon

**Affiliations:** 1 Desmond Tutu TB Centre, Department of Paediatrics and Child Health, Faculty of Medicine and Health Sciences, Stellenbosch University, PO Box 241, 8000 Cape Town, South Africa; 2 Department of Paediatrics, University of Melbourne, Melbourne, Australia; 3 Global TB Program, Department of Pediatrics, Baylor College of Medicine, Houston, USA; 4 Centre for International Child Health, Department of Paediatrics, Imperial College London, Norfolk Place, London, W2 1PG UK

**Keywords:** Tuberculosis, Children, Resistance, Infection, Disease

## Abstract

**Background:**

The relative fitness of organisms causing drug-susceptible (DS) and multidrug-resistant (MDR) tuberculosis (TB) is unclear. We compared the risk of TB infection and TB disease in young child household contacts of adults with confirmed DS-TB and MDR-TB.

**Methods:**

In this cross-sectional analysis we included data from two community-based contact cohort investigation studies conducted in parallel in Cape Town, South Africa. Children <5 years of age with household exposure to an infectious TB case were included between August 2008 to June 2011. Children completed investigation for TB infection (tuberculin skin test) and TB disease (symptom evaluation, chest radiograph, bacteriology) in both studies using standard approaches. The impact of MDR-TB exposure on each covariate of TB infection and TB disease was assessed using univariable and multivariable logistic regression.

**Results:**

Of 538 children included, 312 had DS-TB and 226 had MDR-TB exposure. 107 children with DS-TB exposure had TB infection (34.3%) vs. 101 (44.7%) of children with MDR-TB exposure (adjusted Odds Ratio [aOR]: 2.05; 95% confidence interval [CI]: 1.34–3.12). A total of 15 (6.6%) MDR-TB vs. 27 (8.7%) DS-TB child contacts had TB disease at enrolment (aOR: 0.43; 95% CI: 0.19–0.97).

**Conclusions:**

Our results suggest a higher risk of TB infection in child contacts with household MDR-TB vs. DS-TB exposure, but a lower risk of TB disease. Although potentially affected by residual confounding or selection bias, our results are consistent with the hypothesis of impaired virulence in MDR-TB strains in this setting.

## Background

In 1953, Middlebrook and Cohn demonstrated that mycobacteria resistant to isoniazid were less able to cause disease in guinea pigs than susceptible organisms [1]. More recently, in the laboratory, rifampicin-resistant organisms have been demonstrated to be less able to grow in competition assays than rifampicin-susceptible organisms [2]. However, with time, the rifampicin-resistant organisms frequently develop compensatory mutations that restore fitness [3]. It is unclear how long these compensatory mutations would take to develop in a clinical context. This situation is complicated by differences in virulence between different mycobacterial strains [4, 5] and an association between certain strains and drug resistance [6, 7].

It is important to define “fitness” from the organism’s perspective. To reproduce, *Mycobacterium tuberculosis* must first be transmitted to a new host (requiring ‘infectivity’) and then cause disease (requiring ‘virulence’). The probability of causing tuberculosis (TB) infection in a contact is a function of the infectivity of the organism, the infectiousness of the source case (bacillary load), the duration and intensity of the exposure, and the innate and adaptive immune response of the exposed contact. For TB source cases with disease caused by drug-resistant *M. tuberculosis*, delayed diagnosis and long courses of inadequate treatment may lead to progression of disease severity and more infectious TB, leading to prolonged periods of exposure in their contacts. The probability of causing disease once infected is however a function of the virulence of the organism and is also affected by host factors including age, immunity, the use of effective preventive therapy and other factors.

The issue of infectivity and virulence in the context of drug-resistant (DR)-TB is important as it is central to our understanding of the evolution of the DR-TB epidemic. Mathematical models that assume a fixed fitness cost predict that DR-TB will remain a localised problem and will not evolve into a global epidemic [8]. Models that include heterogeneity in levels of fitness, created by compensatory mutations, however, suggest that the DR-TB epidemic will evolve globally unless specific interventions are employed [9]. We evaluated the impact of drug resistance on the infectivity and virulence of *M. tuberculosis* by determining the risk of TB infection and TB disease in young child household contacts of DR-TB and drug-susceptible (DS)-TB source cases soon after the diagnosis of TB in the identified source case.

## Methods

### Data sources

We completed secondary analysis from two complementary observational cohort household contact investigation studies conducted in parallel in Cape Town, Western Cape, South Africa. The study population consisted of children of black African (mainly Xhosa ethnicity) and mixed race (an ethnic group of mixed ancestry) from urban communities in the Cape Town metropolitan area. The overall TB notification rate in the Cape metropolitan area was 928/100,000 in 2011 (Western Cape Department of Health). Bacillus Calmette-Guérin (BCG) vaccine was routinely given at birth.

Data were obtained from children with household TB exposure to a case of infectious pulmonary DS-TB or MDR-TB (defined as disease caused by *M. tuberculosis* resistant to isoniazid and rifampicin), who were routinely diagnosed and treated at the local TB facility, and were enrolled in two contact investigation studies implemented between August 2008 and June 2011. All source cases were sputum culture-positive for *M. tuberculosis* and had DST completed. Source cases were classified as sputum acid-fast bacilli smear-positive (smear-positive) or smear-negative. Primary study findings of the two cohort studies have been previously reported [10, 11]. Children were eligible for inclusion in this analysis if they were younger than five years old and had an available Mantoux tuberculin skin test (TST) result.

Children with DS-TB exposure were recruited through household contact investigation in three high-burden TB communities (Ravensmead, Uitsig and Khayelitsha), while children with MDR-TB exposure were enrolled through household contact investigations from Khayelitsha, and from a paediatric outpatient clinic at the provincial Tygerberg Hospital, which supports clinical services to children exposed to MDR-TB from communities in the larger metropolitan Cape Town area. Children were recruited by different clinical teams from the same research group. All children were evaluated using standardised techniques and data were captured using standardised forms and tools. All adult TB source cases were identified by local TB clinics and had confirmed pulmonary TB. Routine bacteriological investigation on sputum including sputum smear microscopy (auramine) using standard World Health Organization guidelines [12] and genotypic DST were completed as per local guidelines. Mycobacterial liquid culture was completed using the MGIT 960 system, Becton Dickinson, Sparks, MD, USA and DST completed using the commercial Line Probe Assay (LPA; GenoType® MTBDR*plus*; Hain Lifescience, Nehren, Germany). MDR-TB index cases had resistance to both rifampicin and isoniazid detected on LPA. DS-TB cases enrolled had been initiated on TB treatment during the preceding 3 months, and MDR-TB cases during the preceding 6 months.

### Investigations

Children were classified as being a household contact based on a definition of household which was developed and validated in the study setting, namely all dwellings on the same plot of land that share the same residential address [13]. All children were investigated for TB infection and TB disease status in the same manner using standard protocols. TST was completed by injecting two tuberculin units intradermally (purified protein derivate RT23, Statens Serum Institute), read at 48–72 h. TSTs were placed and read by trained research nurses using the caliper method. A diameter of ≥10 mm was considered positive in HIV-negative and ≥5 mm in HIV-positive children. All children with negative or unknown HIV status were tested for HIV using the Determine™ HIV-1/2 (Abbott Laboratories, Il, USA). Positive or indeterminate HIV rapid tests were confirmed with ELISA (children >18 months) or DNA polymerase chain reaction (PCR; children <18 months).

Standard clinical examination and chest radiography (anteroposterior and lateral) was completed in all children and gastric aspirates completed as clinically indicated (i.e., in the presence of symptoms suggestive of TB or suggestive chest radiograph findings). TB microbiological investigation was completed as described for adult source cases. TB disease in child contacts was defined as confirmed, probable or suspected, using a standard case protocol definition for use in contact investigation studies [14].

### Clinical care

Children diagnosed with DS- or MDR-TB disease were treated based on national and international guidelines. Following exclusion of TB disease, children exposed to MDR-TB were prescribed a preventive therapy regimen based on a local standard of care regimen, consisting of ofloxacin, high-dose isoniazid and ethambutol [15]. Children exposed to DS-TB were referred to routine health services for a standard 6 month course of daily isoniazid.

### Data collection

For children enrolled, information was collected from parents and caregivers on multiple clinical and social factors. Relevant TB medical history was collected from adult TB source cases and verified from clinic-based TB treatment registers and medical records. Data were also collected on household socio-economic status using an asset-based questionnaire from which an index variable was derived from data on reported household assets, with one point awarded for each of a radio, a television, a refrigerator, a cell phone, a phone landline, or a bicycle, and five points for a car, for a total of 11 possible points. This was subsequently categorised into “low” or “high” based on the observed median value.

### Statistical analysis

The datasets from the two studies were combined and relevant variables extracted. Exposures of interest for each outcome were identified prior to analysis using Directed Acyclic Graphs, a method for identifying potential confounders and effect modifiers a priori. Variables identified as potentially relevant for the child’s infection status included ethnicity, age, gender, the MDR-TB status of the adult, whether the child and the adult slept in the same room, the child’s BCG vaccination status (scar and/or documentation on immunization card), child’s previous TB treatment history, reported household tobacco smoking exposure, underweight (as measured by weight for age z-score), HIV status, and household socioeconomic status. The smear status of the adult was also identified as a likely determinant of infection status in the child. However as smear status is largely a result of MDR-TB status, this variable was excluded from the multivariable model for infection. Variables identified as relevant to the child’s disease status among the subset of children with TB infection included age, HIV status, BCG vaccination status, underweight, household tobacco smoke exposure, and the MDR-TB status of the adult.

**Fig. 1:**
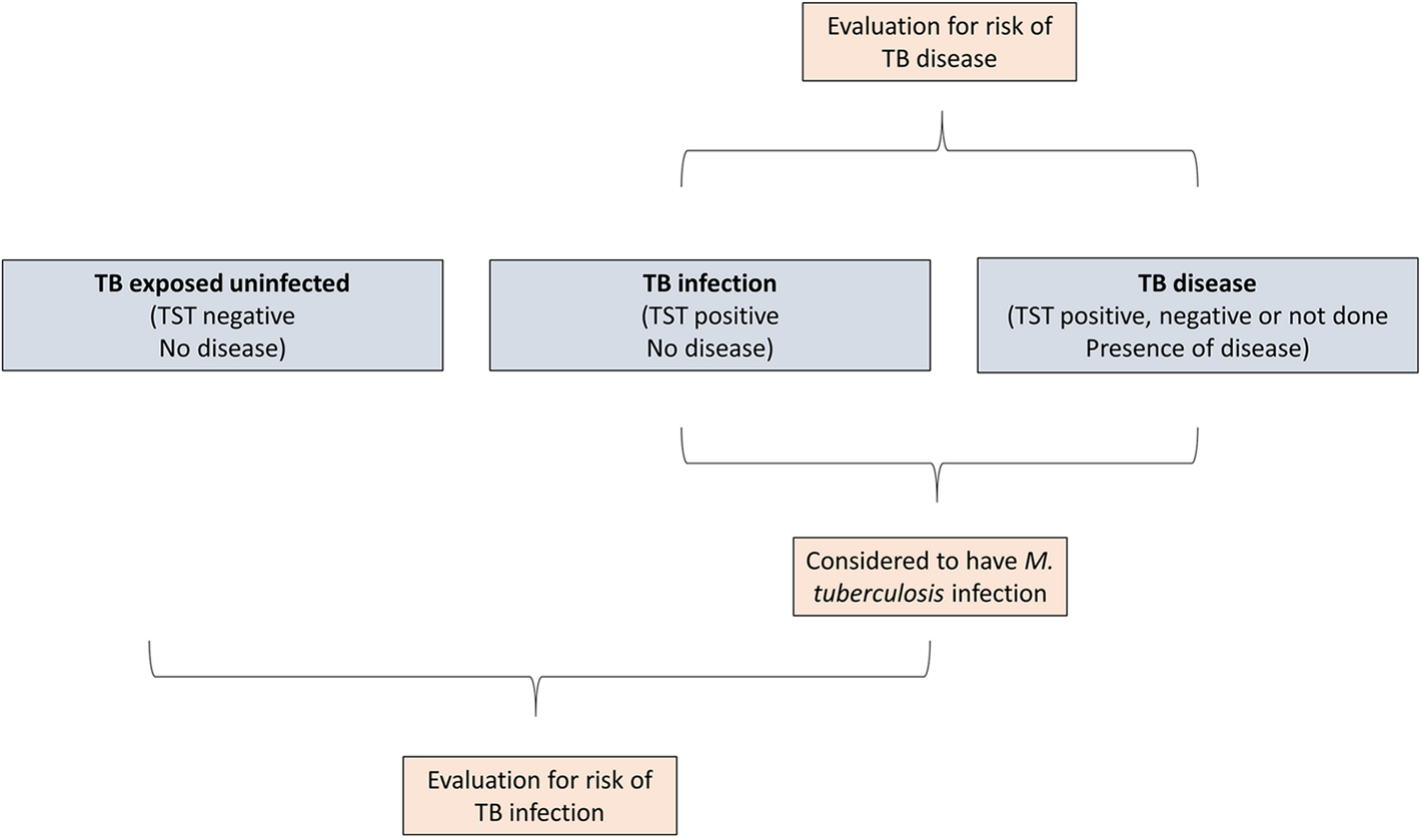
Representation of statistical analysis and classification of cases into TB exposed uninfected, TB infection and TB disease groups

Children were categorised as being TB exposed uninfected (no TB disease and negative TST), TB infected (no TB disease and positive TST) or TB diseased. For the category of TB disease we included children who had confirmed, probable and suspected TB disease. The two main outcomes of interest were TB infection and TB disease. Analysis of risk factors for TB infection compared TB exposed uninfected children to those with TB infection and TB disease, using the variables described above. Analysis of risk factors for TB disease was restricted to children with either TB infection or TB disease (Fig. 1). The statistical significance of baseline differences in relevant covariates according to whether the child was exposed to DS-TB vs. MDR-TB, and to outcome status, were assessed using chi-squared tests. The impact of MDR-TB exposure and of each covariate on both TB infection and TB disease status was assessed using univariable and multivariable logistic regression. Analyses were performed in Stata 13 (StataCorp, Texas, USA). Due to the small number of children with HIV, we performed sensitivity analyses restricted to HIV-negative children to assess whether small cell sizes substantially affected results. We also performed a sensitivity analysis to ascertain whether household-level clustering had any material impact on the results, as some children shared the same adult contact.

## Results

The sample analysed consisted of 316 (58.0%) children from the DS-TB cohort and 229 (42.0%) from the MDR-TB cohort studies (total *n* = 545). Children had a median age of 2 years 7 months (inter-quartile range (IQR) 15 months, 3 years 8 months), and were predominantly of mixed race ethnicity (*n* = 388, 71.2%). Most other children were of black African ethnicity (*n* = 153, 28.0%); one child was European, two Indian, and one “other”. Nine children (1.7%) were HIV-positive. MDR-TB child contacts differed from DS-TB contacts regarding several relevant covariates (Table 1): they were more likely to be of black ethnicity (44.1% vs. 16.5%; *p* < 0.001), more likely to be HIV-positive (3.7% vs. 0.3%; *p* < 0.01), were less likely to have a BCG scar or documented BCG vaccination (81.2% vs. 98.1%; *p* < 0.001), were more likely to have had previous TB treatment (9.2% vs. 2.5%; *p* < 0.01), and were less likely to have been exposed to household tobacco smoke (63.3% vs. 80.4%; *p* < 0.001).

**Table 1:** Baseline characteristics in children with household multidrug-resistant tuberculosis and drug-susceptible tuberculosis exposure

Risk factors and clinical states	DS-TB exposure (*n* = 316) N (%)	MDR-TB exposure (*n* = 229) N (%)
Child factors		
< 1 year	48 (15.2)	50 (21.8)
1 year	66 (20.9)	41 (17.9)
2 years	71 (22.5)	44 (19.2)
3 years	73 (23.1)	56 (24.5)
4 years	58 (18.4)	38 (16.6)
Male	162 (51.3)	119 (52.2)
Black African (vs. mixed race)	52 (16.5)	101 (44.1)**
HIV-positive	1 (0.3)	8 (3.7)*
BCG scar/vaccination documented	310 (98.1)	181 (81.2)**
Previous tuberculosis treatment	8 (2.5)	21 (9.2)*
Weight for age (z-score) < −2	32 (10.1)	23 (10.1)
Sleeps in same room as TB source case	79 (25.3)	34 (15.0)**
Sleeps in same bed as TB source case	20 (6.4)	57 (25.2)**
Adult source case /household factors		
Source case sputum acid-fast bacilli smear-positive	181 (62.9)	180 (80.0)**
Household tobacco smoke exposure	245 (80.4)	145 (63.3)**
Mean socioeconomic index (x/11), n (standard deviation)	4.0 (2.6)	4.1 (2.5)
Clinical states		
Exposure no infection	205 (65.7)	125 (61.3)
Infection no disease	80 (25.6)	86 (38.1)*
Disease	27 (8.7)	15 (6.6)

*p* < 0.011*; *p* < 0.001**

*MDR-TB Mycobacterium tuberculosis* resistant to rifampicin and isoniazid (defined by line probe assay)

*DS-TB Mycobacterium tuberculosis* susceptible to rifampicin and isoniazid (defined by line probe assay)

TST results were missing in four children in the DS-TB group and in six children in the MDR-TB group. These were excluded from analyses unless they had TB disease (used as a proxy for TB infection). Three children in the MDR-TB-exposed group were diagnosed with TB disease but did not have a TST result available, and were included. Therefore 538 of 545 children had outcome data available. Among child contacts of DS-TB source cases, 34.3% of children (107/312) had evidence of TB infection compared to 44.7% of contacts of MDR-TB source cases (101/226). Twenty seven of 312 child DS-TB contacts (8.7%) had TB disease (seven were confirmed) compared to 15 (of 226) MDR-TB contacts (6.6%) who had disease (three were confirmed).

**Fig. 2:**
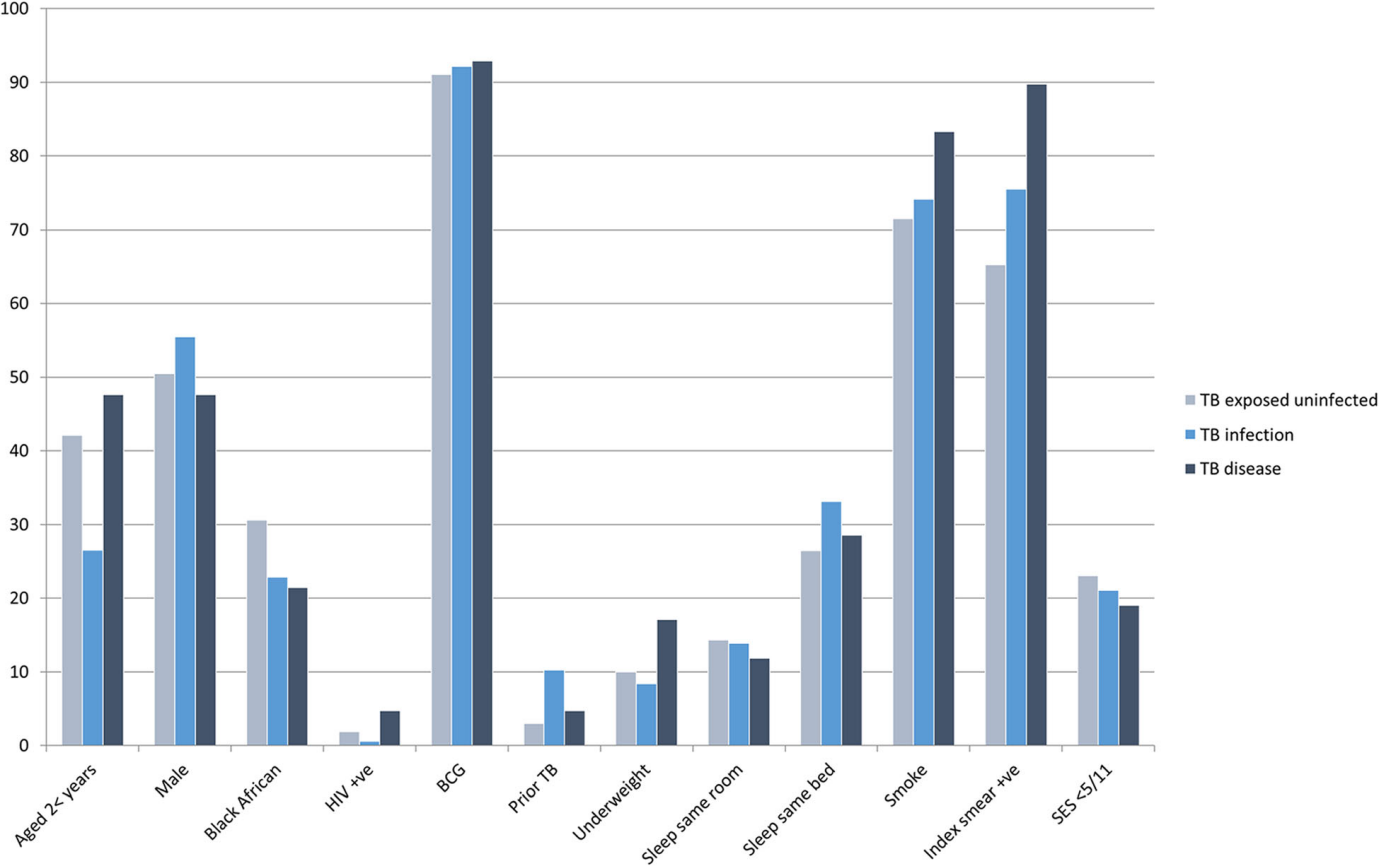
Baseline characteristics stratified by TB exposed uninfected, TB infection and TB disease status

In univariable analysis children with TB infection were more likely to be older than two years, more likely to have had previous TB treatment, and more likely to sleep in the same bed as the adult TB source case compared to TB exposed uninfected children (Table 2; Fig. 2). Children with TB disease were more likely to be younger than two years, less likely to be of black African ethnicity, more likely to be underweight-for-age, and more likely to have household tobacco smoke exposure (Table 3).

**Table 2:** Characteristics associated with tuberculosis infection status in child contacts of drug-susceptible tuberculosis and multidrug-resistant tuberculosis

	Total	Infected N (%)	OR (95%CI)	AOR (95% CI)
Child factors				
Age < 2 years	203	64 (31.5%)	ref	ref
Age ≥ 2 years	335	144 (43%)	1.64 (1.13, 2.36)	1.61 (1.09, 2.37)
Female	259	96 (37.1%)	ref	ref
Male	278	112 (40.3%)	1.15 (0.81, 1.62)	1.02 (0.70, 1.47)
Mixed race / other ethnicity	390	161 (41.3%)	ref	ref
Black African ethnicity	148	47 (31.8%)	0.66 (0.44, 0.99)	0.54 (0.33, 0.88)
HIV-negative	518	202 (39%)	ref	ref
HIV-positive	9	3 (33.3%)	0.78 (0.19, 3.16)	0.66 (0.15, 2.94)
No BCG scar /no vaccination documented	45	16 (35.6%)	ref	ref
BCG scar/vaccination documented	487	191 (39.2%)	1.17 (0.62, 2.21)	1.62 (0.80, 3.29)
No prior tuberculosis treatment (disease)	509	189 (37.1%)	ref	ref
Prior tuberculosis treatment (disease)	29	19 (65.5%)	3.22 (1.47, 7.06)	2.36 (1.03, 5.39)
Weight for age (z-score) ≥ −2	482	186 (38.6%)	ref	ref
Weight for age (z-score) < −2	54	21 (38.9%)	1.01 (0.57, 1.80)	1.04 (0.56, 1.92)
Sleeps in different room to TB source case	348	132 (37.9%)	ref	ref
Sleeps in same room as TB source case	113	40 (35.4%)	0.90 (0.58, 1.40)	0.85 (0.53, 1.36)
Sleeps in same bed as TB source case	77	36 (46.8%)	1.44 (0.87, 2.36)	1.05 (0.60, 1.82)
Adult source case/household factors				
DS-TB source case	312	107 (34.3%)	ref	ref
MDR-TB source case	226	101 (44.7%)	1.55 (1.09, 2.20)	2.05 (1.34, 3.12)
No household tobacco smoke exposure	144	50 (34.7%)	ref	ref
Household tobacco smoke exposure	394	158 (40.1%)	1.26 (0.85, 1.87)	1.22 (0.76, 1.94)
Socioeconomic index ≥ 5/11	419	165 (39.4%)	ref	ref
Socioeconomic index <5/11	119	43 (36.1%)	0.87 (0.57, 1.33)	0.79 (0.51, 1.25)

*MDR-TB Mycobacterium tuberculosis* resistant to rifampicin and isoniazid (defined by line probe assay)

*DS-TB Mycobacterium tuberculosis* susceptible to rifampicin and isoniazid (defined by line probe assay)

**Table 3:** Characteristics associated with tuberculosis disease in child contacts of drug-susceptible tuberculosis and multidrug-resistant tuberculosis, among children with tuberculosis infection

	Total	Disease N (%)	OR (95% CI)	AOR (95% CI)
Child factors				
Age < 2 years	64	20 (31.3%)	ref	ref
Age ≥ 2 years	144	22 (15.3%)	0.40 (0.20, 0.80)	0.32 (0.15, 0.71)
HIV negative	202	40 (19.8%)	ref	ref
HIV positive	3	2 (66.7%)	8.10 (0.72, 91.57)	23.0 (1.11, 477.86)
No BCG scar / no vaccination documented	16	3 (18.8%)	ref	ref
BCG scar / vaccination documented	191	39 (20.4%)	1.11 (0.30, 4.09)	1.14 (0.21, 6.20)
Weight for age (z-score) ≥ −2	186	34 (18.3%)	ref	ref
Weight for age (z-score) < −2	21	7 (33.3%)	2.24 (0.84, 5.96)	2.17 (0.72, 6.55)
Adult source case factors				
DS-TB	107	27 (25.2%)	ref	ref
MDR-TB	101	15 (14.9%)	0.52 (0.26, 1.04)	0.43 (0.19, 0.97)
Smear-negative source case	43	4 (9.3%)	ref	ref
Smear-positive source case	155	35 (22.6%)	2.84 (0.95, 8.51)	4.24 (1.31, 13.78)

*MDR-TB Mycobacterium tuberculosis* resistant to rifampicin and isoniazid (defined by line probe assay)

*DS-TB Mycobacterium tuberculosis* susceptible to rifampicin and isoniazid (defined by line probe assay)

In the multivariable model for TB infection, age older than two years was associated with higher odds of having TB infection (Table 2), as was exposure to a source case with MDR-TB (adjusted odds ratio [aOR]: 2.05; 95% confidence interval [CI]: 1.34–3.12). Children of black African ethnicity were less likely to be TB-infected compared to children of mixed race or other ethnicities.

In the multivariable model for TB disease, age older than two years was protective against TB disease (Table 3). Being exposed to MDR-TB was associated with a lower odds of TB disease (aOR: 0.43; 95%CI: 0.19–0.97). HIV-positive children had markedly higher odds of having TB disease compared to HIV-negative children. Restricting the analysis to HIV-negative children did not substantially affect the results for TB disease. Although the precision of the estimates for HIV was low, HIV infection was retained in the final model as HIV status was a key determinant of TB disease. The sensitivity analysis accounting for clustering did not materially change the results for either TB infection or TB disease.

## Discussion

In this study we present a cross-sectional analysis comparing baseline data from two large cohorts of young children with documented household exposure to infectious TB, one group exposed to DS-TB and the other to MDR-TB, from the same study setting and investigated using standardised protocols. We found that a higher proportion of children exposed to MDR-TB had evidence of TB infection when enrolled but that a lower proportion had TB disease. Children of mixed race ethnicity were at increased risk of TB infection as were older children. Children with HIV infection were at an increased risk of having TB disease, as were younger children. Overall, our results are consistent with data from other studies. Systematic reviews of the yield from household contact investigations demonstrate similar proportions of child contacts having prevalent TB infection and TB disease in both DS-TB [16] and MDR-TB contacts [17].

The relation between age, HIV status and the risk of TB infection and disease is well established in children [18, 19]. As children become older there is an increasing probability of TST positivity due to accumulated age-related TB exposure [20]. It is also possible that the reduced sensitivity of the TST in younger children may lead to false negatives, with fewer children being diagnosed with TB infection. However, we have found limited evidence for this effect in a large contact investigation in our setting [10]. False positive TST status in young children (<2 years of age) due to BCG effect could have led to more young children being TST positive, which was not observed in our study. Young age and HIV infection status are both well recognised risk factors for progressing to TB disease following *M. tuberculosis* infection, given the developing immune system and the impact of T-cell immunological suppression, respectively [21]. Our study again highlights the vulnerability of these children, who are in most need of early identification and preventive therapy before they progress from infection to disease [18, 22].

There was a clear influence of drug resistance on the risk of TB infection and disease in young children in our study. While these results could be, to some extent, the result of residual confounding, they are consistent with findings from other studies and also with plausible biological mechanisms. Children exposed to MDR-TB source cases are likely to have been exposed for prolonged periods of time and to more severe (and consequently infectious) TB disease in the source case, due to diagnostic and treatment delay in adults with MDR-TB [23]. That fewer children had progressed to TB disease at screening may reflect different populations and socioeconomic circumstances associated with MDR-TB households vs. DS-TB households. However, it is possible that this reduced prevalence of TB disease in children exposed to MDR-TB reflects reduced virulence of resistant mycobacteria.

A number of epidemiological studies have observed no differences in the risk of TB infection and/or disease between contacts exposed to DR-TB and DS-TB. Barrosso and colleagues carried out a case-control study in Brazil between 1990 and 1999. They evaluated the contacts of 126 patients with MDR-TB and 176 with DS-TB and found similar numbers of contacts per source case between the two groups. They also found similar proportions of contacts who went on to develop incident TB disease [24]. Snider and colleagues compared the risk of infection in contacts of nearly four hundred DR-TB cases to the risk in contacts of a similar number of matched DS-TB cases. This study was carried out in the USA from 1975 to 1977. The DR-TB cases had disease caused by strains of *M tuberculosis* resistant to isoniazid and/or streptomycin, and household childhood contacts (<15 years) were evaluated for evidence of TST positivity. No difference in TST positivity was found between the two groups of contacts [25]. Teixeira and colleagues evaluated contacts of MDR-TB and DS-TB for evidence of TB infection and TB disease. This study was conducted in Brazil between 1994 and 1998. Amongst 157 household contacts of 26 MDR-TB cases there was no difference in risk of TB infection or prevalent TB disease, as compared to 251 contacts of 52 DS-TB cases [26]. In contrast to these studies, Grandjean and colleagues recently carried out a study in Peru which demonstrated that adult and child household contacts of MDR-TB patients were only half as likely to develop incident TB disease compared to contacts of DS-TB [27]. An observational study from India found that contacts of isoniazid-resistant TB were more likely to have TB infection compared to contacts of DS-TB, but that on follow-up, both groups had similar rates of incident TB [28]. A study from Romania demonstrated lower rates of TB infection and prevalent disease in child household contacts of patients with isoniazid-resistant TB disease compared to DS-TB [29]. Our results add to this body of work and are consistent with some of the previous studies but are different to others. Differences between studies may be a result of differing research methodologies. Although approaches were relatively similar between the different studies described above, differences in duration of exposure, and definitions of infection and disease, varied to some degree. Some studies evaluated prevalent TB disease while others incident disease in those found to be disease-free at baseline. Differences may be the result of urban vs. rural settings, the presence of HIV infection, the degree of poverty, the health systems in which children were evaluated, or that studies were conducted in different time periods. For paediatric TB, the likelihood of a confirmed diagnosis is impacted, to some extent, by how thoroughly a child is investigated. Some settings may have investigated drug-resistant contacts more extensively than those exposed to drug-susceptible source cases. Conversely, due to the toxicity and duration of MDR-TB treatment in children, clinicians may have been more willing to clinically diagnose children with TB disease if exposed to a drug-susceptible case than than to an MDR-TB case. Finally genetic differences between host and pathogen populations may also have influenced rates of infection and disease. The relationship between train types, resistance and virulence is complex and influenced by geography [4–7]. To comprehensively address this question, a prospective, multi-site study could evaluate child contacts of drug-resistant and drug-susceptible TB source cases, with children investigated by study teams trained using standardised methodology in real-time. Ideally, those investigating the children would be blinded to exposure.

Our study has a number of limitations. First, data analyses were cross-sectional. We evaluated children at the first visit following identification of the TB source case, and this analysis assessed prevalent TB infection and disease only. For TST, we acknowledge that a small proportion of cases will test negative at initial screening (if seen very early before immunological memory has been established) but may subsequently convert their TST. Second, we acknowledge that TST is an imperfect measure of TB infection, especially in young and HIV-positive children, where interferon-gamma release assays (IGRAs) have improved sensitivity and specificity [10, 30]. We did not consider IGRA results in this analysis, as they were not available for the MDR-TB cohort. We also acknowledge that previous TB disease, BCG vaccination, and non-tuberculous mycobacterial infection may all have led to a positive TST result. It is also possible that children may have had undocumented exposure to other mycobacterial strains (i.e. outside of documented household exposure); this would however not be expected to be different between DS- and MDR-TB exposed children aged below five years of age. Third, we did not report on incident TB during follow-up. Fourth, the two studies were conducted separately but in parallel. Although separate clinical teams conducted the two studies, both were conducted by the same centre and research personnel were trained in a standard manner. Prior to study implementation, we standardised study practices with consistent training, data collection tools, TB investigations and case definitions for TB infection and disease. However, in the DS-TB cohort, children were recruited if they had been exposed in the preceding three months to an infectious source case, whereas in the MDR-TB cohort a six month cut-off was used. If this had caused selection bias, however, it would have served to attenuate any negative association between MDR-TB exposure and TB disease rather than exaggerate it, as children in the MDR-TB cohort had twice as long to develop TB before the baseline assessment. Nonetheless, we saw a substantially lower prevalence of disease among the MDR-TB cohort. It is possible that the differing exposure cut-off may have biased the TB infection analysis towards an increased effect estimate in the MDR-TB exposed cohort. There is an additional possibility of bias in the MDR-TB child contacts due to enrolment of some children at hospital, compared to the community-based recruitment strategy for DS-TB contacts. The recruitment catchment areas, although overlapping and drawing patients from similar communities, were not identical – this is reflected in more black African children in the MDR-TB group and also a higher proportion of children with HIV infection and a lower proportion with evidence of BCG vaccination. The prevalence of DR-TB in the catchment areas, however, are unlikely to be different given the results of previous DST surveys which showed similar rates of DR-TB between children from Tygerberg Hospital and surrounding communities [31]. A further potential source of bias was that clinicians were not blinded to the DST pattern of the source case. Even with the use of standardised protocols and TB disease definitions, it is possible that clinicians may have been more willing to make a diagnosis of TB disease in children exposed to DS-TB due to shorter and less toxic treatment.

## Conclusions

We demonstrate that children exposed to MDR-TB are more likely to have a positive test of TB infection than children exposed to DS-TB but are less likely to present with prevalent TB disease. Although potentially affected by residual confounding or selection bias, our results are consistent with the hypothesis of impaired virulence in MDR-TB strains in this setting.

## Abbreviations

aOR: Adjusted odds ratio
BCG: Bacillus Calmette-Guérin
cART: Combination antiretroviral therapy
CI: Confidence interval
DR: Drug-resistant
DS: Drug-susceptible
DST: Drug susceptibility testing
HIV: Human immunodeficiency virus
IGRA: Interferon-gamma release assay
IQR: Inter-quartile range
LPA: Line probe assay
*M. tuberculosis*: *Mycobacterium tuberculosis*
MDR: Multidrug-resistant
OR: Odds Ratio
PCR: Polymerase chain reaction
TB: Tuberculosis
TST: Tuberculin skin test

## References

[1] Middlebrook G, Cohn ML (1953). Some observations on the pathogenicity of isoniazid-resistant variants of tubercle bacilli. Science.

[2] Gagneux S, Long CD, Small PM, Van T, Schoolnik GK, Bohannan BJ (2006). The competitive cost of antibiotic resistance in Mycobacterium tuberculosis. Science.

[3] Comas I, Borrell S, Roetzer A, Rose G, Malla B, Kato-Maeda M, Galagan J, Niemann S, Gagneux S (2012). Whole-genome sequencing of rifampicin-resistant Mycobacterium tuberculosis strains identifies compensatory mutations in RNA polymerase genes. Nat Genet.

[4] Hanekom M, van der Spuy GD, Streicher E, Ndabambi SL, McEvoy CR, Kidd M, Beyers N, Victor TC, van Helden PD, Warren RM (2007). A recently evolved sublineage of the Mycobacterium tuberculosis Beijing strain family is associated with an increased ability to spread and cause disease. J Clin Microbiol.

[5] Thwaites G, Caws M, Chau TT, D'Sa A, Lan NT, Huyen MN, Gagneux S, Anh PT, Tho DQ, Torok E (2008). Relationship between Mycobacterium tuberculosis genotype and the clinical phenotype of pulmonary and meningeal tuberculosis. J Clin Microbiol.

[6] Johnson R, Warren RM, van der Spuy GD, Gey van Pittius NC, Theron D, Streicher EM, Bosman M, Coetzee GJ, van Helden PD, Victor TC (2010). Drug-resistant tuberculosis epidemic in the Western Cape driven by a virulent Beijing genotype strain. Int J Tuberc Lung Dis.

[7] Grandjean L, Iwamoto T, Lithgow A, Gilman RH, Arikawa K, Nakanishi N, Martin L, Castillo E, Alarcon V, Coronel J (2015). The Association between Mycobacterium tuberculosis genotype and drug resistance in Peru. PLoS One.

[8] Dye C, Espinal MA (2001). Will tuberculosis become resistant to all antibiotics?. Proc Biol Sci.

[9] Cohen T, Murray M (2004). Modeling epidemics of multidrug-resistant M. tuberculosis of heterogeneous fitness. Nat Med.

[10] Mandalakas AM, Kirchner HL, Walzl G, Gie RP, Schaaf HS, Cotton MF, Grewal HM, Hesseling AC (2015). Optimizing the detection of recent tuberculosis infection in children in a high tuberculosis-HIV burden setting. Am J Respir Crit Care Med.

[11] Seddon JA, Hesseling AC, Godfrey-Faussett P, Fielding K, Schaaf HS (2013). Risk factors for infection and disease in child contacts of multidrug-resistant tuberculosis: a cross-sectional study. BMC Infect Dis.

[12] World Health Organization G, Switzerland,: Laboratory diagnosis of tuberculosis by sputum microscopy: the handbook. 2013. Available at: http://www.stoptb.org/wg/gli/assets/documents/TB%20MICROSCOPY%20HANDBOOK_FINAL.pdf. Accessed 8 Dec 2016.

[13] Van Wyk SS, Mandalakas AM, Enarson DA, Gie RP, Beyers N, Hesseling AC (2012). Tuberculosis contact investigation in a high-burden setting: house or household?. Int J Tuberc Lung Dis.

[14] Wiseman CA, Mandalakas AM, Kirchner HL, Gie RP, Schaaf HS, Walters E, Hesseling AC (2015). Novel application of NIH case definitions in a paediatric tuberculosis contact investigation study. Int J Tuberc Lung Dis.

[15] Seddon JA, Hesseling AC, Finlayson H, Fielding K, Cox H, Hughes J, Godfrey-Faussett P, Schaaf HS (2013). Preventive therapy for child contacts of multidrug-resistant tuberculosis: a prospective cohort study. Clin Infect Dis.

[16] Morrison J, Pai M, Hopewell PC (2008). Tuberculosis and latent tuberculosis infection in close contacts of people with pulmonary tuberculosis in low-income and middle-income countries: a systematic review and meta-analysis. Lancet Infect Dis.

[17] Shah NS, Yuen CM, Heo M, Tolman AW, Becerra MC (2014). Yield of contact investigations in households of patients with drug-resistant tuberculosis: systematic review and meta-analysis. Clin Infect Dis.

[18] Marais BJ, Gie RP, Schaaf HS, Hesseling AC, Obihara CC, Starke JJ, Enarson DA, Donald PR, Beyers N (2004). The natural history of childhood intra-thoracic tuberculosis: a critical review of literature from the pre-chemotherapy era. Int J Tuberc Lung Dis.

[19] Dodd PJ, Seddon JA (2015). Understanding the contribution of HIV to the risk of developing tuberculosis in children: a systematic review and meta-analysis. Int J Tuberc Lung Dis.

[20] Wood R, Liang H, Wu H, Middelkoop K, Oni T, Rangaka MX, Wilkinson RJ, Bekker LG, Lawn SD (2010). Changing prevalence of tuberculosis infection with increasing age in high-burden townships in South Africa. Int J Tuberc Lung Dis.

[21] Basu Roy R, Whittaker E, Kampmann B (2012). Current understanding of the immune response to tuberculosis in children. Curr Opin Infect Dis.

[22] Bhat GJ, Diwan VK, Chintu C, Kabika M, Masona J (1993). HIV, BCG and TB in children: a case control study in Lusaka, Zambia. J Trop Pediatr.

[23] Naidoo P, van Niekerk M, du Toit E, Beyers N, Leon N (2015). Pathways to multidrug-resistant tuberculosis diagnosis and treatment initiation: a qualitative comparison of patients’ experiences in the era of rapid molecular diagnostic tests. BMC Health Serv Res.

[24] Barroso E, Mota R, Pinheiro V, Campelo C, Rodrigues J (2004). Occurence of active tuberculosis in households inhabited by patients with susceptible and mulidrug-resistant tuberculosis. J Bras Pneumol.

[25] Snider DE, Jr., Kelly GD, Cauthen GM, Thompson NJ, Kilburn JO: Infection and disease among contacts of tuberculosis cases with drug-resistant and drug-susceptible bacilli. Am Rev Respir Dis 1985;132(1):125–132. 10.1164/arrd.1985.132.1.125 3925826

[26] Teixeira L, Perkins MD, Johnson JL, Keller R, Palaci M, do Valle Dettoni V, Canedo Rocha LM, Debanne S, Talbot E, Dietze R (2001). Infection and disease among household contacts of patients with multidrug-resistant tuberculosis. Int J Tuberc Lung Dis.

[27] Grandjean L, Gilman RH, Martin L, Soto E, Castro B, Lopez S, Coronel J, Castillo E, Alarcon V, Lopez V (2015). Transmission of multidrug-resistant and drug-susceptible tuberculosis within households: a prospective cohort study. PLoS Med.

[28] Tuberculosis Research Centre, Indian Council of Medical Research (ICMR), Chennai, India. Risk of tuberculosis among contacts of isoniazid-resistant and isoniazid-susceptible cases. Int J Tuberc Lung Dis*.* 2011;15(6):782–788. 10.5588/ijtld.09.0327 21575299

[29] Siminel M, Bungetzianu G, Anastasatu C (1979). The risk of infection and disease in contacts with patients excreting Mycobacterium tuberculosis sensitive and resistant to isoniazid. Bull Int Union Tuberc.

[30] Mandalakas AM, Detjen AK, Hesseling AC, Benedetti A, Menzies D (2011). Interferon-gamma release assays and childhood tuberculosis: systematic review and meta-analysis. Int J Tuberc Lung Dis.

[31] Schaaf HS, Marais BJ, Hesseling AC, Gie RP, Beyers N, Donald PR (2006). Childhood drug-resistant tuberculosis in the Western Cape Province of South Africa. Acta Paediatr.

