# Correction to: The impact of drug resistance on the risk of tuberculosis infection and disease in child household contacts: a cross sectional study

**DOI:** 10.1186/s12879-017-2806-x

**Published:** 2017-11-07

**Authors:** Vera Golla, Kathryn Snow, Anna M. Mandalakas, H. Simon Schaaf, Karen Du Preez, Anneke C. Hesseling, James A. Seddon

**Affiliations:** 1 Desmond Tutu TB Centre, Department of Paediatrics and Child Health, Faculty of Medicine and Health Sciences, Stellenbosch University, Box 241, Cape Town, PO 8000 South Africa; 2 Department of Paediatrics, University of Melbourne, Melbourne, Australia; 3 Global TB Program, Department of Pediatrics, Baylor College of Medicine, Houston, USA; 4 Centre for International Child Health, Department of Paediatrics, Imperial College London, Norfolk Place W2 1PG, London, UK

## Correction

After publication of the original article [1] the authors noted that the following errors had occurred:

- The name of the author H. Simon Schaaf had been incorrectly tagged as Simon H. Schaaf. This has been corrected in the author list above.
- The first *p* value below Table 1 is listed as *p* < 0.011, however it should be *p* < 0.01. An updated version of this table is included with this Correction.

**Table 1:** Baseline characteristics in children with household multidrug-resistant tuberculosis and drug-susceptible tuberculosis exposure

Risk factors and clinical states	DS-TB exposure (*n* = 316) N (%)	MDR-TB exposure (*n* = 229) N (%)
Child factors		
< 1 year	48 (15.2)	50 (21.8)
1 year	66 (20.9)	41 (17.9)
2 years	71 (22.5)	44 (19.2)
3 years	73 (23.1)	56 (24.5)
4 years	58 (18.4)	38 (16.6)
Male	162 (51.3)	119 (52.2)
Black African (vs. mixed race)	52 (16.5)	101 (44.1)^**^
HIV-positive	1 (0.3)	8 (3.7)^*^
BCG scar/vaccination documented	310 (98.1)	181 (81.2)^**^
Previous tuberculosis treatment	8 (2.5)	21 (9.2)^*^
Weight for age (z-score) < −2	32 (10.1)	23 (10.1)
Sleeps in same room as TB source case	79 (25.3)	34 (15.0)^**^
Sleeps in same bed as TB source case	20 (6.4)	57 (25.2)^**^
Adult source case /household factors		
Source case sputum acid-fast bacilli smear-positive	181 (62.9)	180 (80.0)^**^
Household tobacco smoke exposure	245 (80.4)	145 (63.3)^**^
Mean socioeconomic index (x/11), n (standard deviation)	4.0 (2.6)	4.1 (2.5)
Clinical states		
Exposure no infection	205 (65.7)	125 (61.3)
Infection no disease	80 (25.6)	86 (38.1)^*^
Disease	27 (8.7)	15 (6.6)

*MDR-TB Mycobacterium tuberculosis* resistant to rifampicin and isoniazid (defined by line probe assay)

*DS-TB Mycobacterium tuberculosis* susceptible to rifampicin and isoniazid (defined by line probe assay)

*p* < 0.01^*^; *p* < 0.001^**^

The original article has also been corrected.
